# The Physician as a Business Man

**Published:** 1911-10

**Authors:** 


### The Physician as a Business Man

THE business interests of the physician have been very frankly considered of late years and within a year, a special journal, published in Philadelphia, has been established, solely devoted to them. "Business" for our profession, is included under three general headings: economic problems affecting the mutual relations and general welfare of the entire profession; profitable and convenient conduct of the financial side of an individual practice; wisdom in purchasing, expending and investing, after the doctor has earned—and collected—his money.

In regard to the first topic, it should be emphasised that the genuine welfare of the profession involves no conflict with ethical principles, although it may happen that certain prejudices, based on old customs, etiquette and the like, may be confused with ethics in a formal code and may require alteration to conform to modern conditions.

We cannot refrain from a feeling of local pride that the territory of and contributors to the Buffalo Medical Journal have had much to do with some of these readjustments. For example, the long conflict which disrupted the profession of New York State, which brought the State Society out of affiliation with the American Medical Association, of which it was really the founder and which, indirectly led to state control of medical education, was due in large part to a realisation that refusal to consult with a Homeopath, was not a matter of principle but of prejudice. We must not forget that a broadminded respect for individual views and an elevation of medical standards are primarily, ethical reforms. Neither should we ignore the fact that they are matters of sound business policy which have reunited and strengthened the entire profession.

Only recently, the Medical Society of the County of Erie, at the instigation of Dr. Edward E. Haley, has taken up the matter of Contract Practice, in a radical, practical, spirit, considering both the welfare of the profession, the ultimate interests of the laity—since, in the long run, ten dollars' worth of professional service can not be obtained for a dollar—and general principles of fair play. The time is ripe for such a movement and, already, the profession in other localities is showing an interest in the results expected in the home city of the Buffalo Medical Journal. Just as in the former example, there was liklihood of overlooking the business aspects of the reform, in the latter, there is danger that we shall regard and allow others to regard the reform as purely a matter of business, whereas, it is in the highest degree, one of ethics.

Our attention has been called to the general subject of business, for the medical profession, by two original and an editorial article in the September 1911 issue of the *St. Paul Medical Journal.* Dr. Edwin White, treats of bonds and mortgages; Dr. Webster Wheelock of farm mortgages. The former rather leans toward bonds, on account of the inelasticity of the mortgage in responding to fluctuations of value. Both authors, as well as the editor, warn against the various schemes in which worthless stock is foisted upon an untrained purchaser. There is no doubt but that promotors have extracted millions of hard earned dollars from our profession, which has been regarded as the legitimate prey of mining, plantation and other stock companies, very much as farmers have been considered the special prey of green goods men.

Our advice is not to imagine that hard working, specially skilled business men are going to run a stock company for the sake of investors who know nothing of the business. In a great many instances, the real business of the company is not getting gold, oil, copper, tropical crops, and the like, out of the ground, or manufacturing or retailing goods, but getting your money for a prettily engraved piece of paper. In other cases, the nominal business of the company is actually carried on but the stock receipts are also part of the profits of the manipulators and the stock is merely votes as to how the business shall be managed. In some instances, too, the interest of stock holders is honestly considered and in such companies, the stock is usually above par so that the ordinary investor might as well leave his money in a bank. There are exceptions, but be sure that you really have encountered one before you invest.

We would also advise strongly against investing at a distance. Remember that we naturally envy the other man's lot and while we deny the superstition, instinctively hold to the notion of a land of gold or of one where there is fabulous abundance obtainable with little labor. What Dr. Wheelock says is good advice for the physician who lives near the farm and can watch it, if necessary manage it. What Dr. White says as to the non-participation of mortgage investments in rise of values is true but, if made with a safe margin and for a reasonable time limit, they are also free from the opposite tendency. For one who is a shrewd judge, an out-and-out purchase of tangible property, is, of course better than a mortgage investment and, provided that the property is accessible and actually usable, no great loss need be expected even if visionary hopes of rapid rise in value fail to materialise.

